# The allergy adjuvant effect of particles – genetic factors influence antibody and cytokine responses

**DOI:** 10.1186/1471-2172-6-11

**Published:** 2005-06-21

**Authors:** Unni Cecilie Nygaard, Audun Aase, Martinus Løvik

**Affiliations:** 1 Division of Environmental Medicine, Norwegian Institute of Public Health, P.O.Box 4404 Nydalen, NO-0403 Oslo, Norway; 2 Division of Infectious Disease Control, Norwegian Institute of Public Health, P.O.Box 4404 Nydalen, NO-0403 Oslo, Norway; 3 Institute of Cancer Research and Molecular Medicine, Faculty of Medicine, Norwegian University of Science and Technology, NO-7489 Trondheim, Norway

## Abstract

**Background:**

There is increasing epidemiological and experimental evidence for an aggravating effect of particulate air pollution on asthma and allergic symptoms and, to a lesser extent, on allergic sensitization. Genetic factors appear to influence not only the magnitude, but also the quality of the adjuvant effect of particles with respect to allergen-specific IgE (Th2-associated) and IgG2a (Th1-associated) responses. In the present study, we aimed to investigate how the genetic background influences the responses to the allergen and particles alone and in combination. We examined how polystyrene particles (PSP) affected the IgE and IgG2a responses against the model allergen ovalbumin (OVA), after subcutaneous injection into the footpad of BALB/cA, BALB/cJ, NIH and C3H/HeN mice, Further, *ex vivo *IL-4, IFN-γ and IL-10 cytokine secretion by Con A-stimulated cells from the draining popliteal lymph node (PLN) five days after injection of OVA and PSP separately or in combination was determined.

**Results:**

PSP injected with OVA increased the levels of OVA-specific IgE antibodies in all strains examined. In contrast, the IgG2a levels were significantly increased only in NIH and C3H/HeN mice. PSP in the presence of OVA increased cell numbers and IL-4, IL-10 and IFN-γ levels in BALB/cA, NIH and C3H/HeN mice, with the exception of IFN-γ in NIH mice. However, each mouse strain had their unique pattern of response to OVA+PSP, OVA and PSP, and also their unique background cytokine response (i.e. the cytokine response in cells from mice injected with buffer only).

**Conclusion:**

Genetic factors (i.e. the strain of mice) influenced the susceptibility to the adjuvant effect of PSP on both secondary antibody responses and primary cellular responses in the lymph node, as well as the cellular responses to both OVA and PSP given separately. Interestingly, PSP alone induced cytokine responses in the lymph node in some of the mouse strains. Furthermore, we found that the *ex vivo *cytokine patterns did not predict the *in vivo *Th2- and Th1-associated antibody response patterns in the different mouse strains. The results indicate that insoluble particles act by increasing the inherent response to the allergen, and that the genetic background may determine whether an additional Th1-associated component is added to the response.

## Background

Numerous epidemiological studies conducted in different parts of the world have demonstrated a consistent association between levels of ambient air particles and various health outcomes, including mortality, cardiopulmonary disease, reduced lung function, and exacerbation of asthma and allergy-related symptoms (reviewed in [1,2]). Some populations seem to be at particular risk, like the elderly, children or patients with diabetes, chronic obstructive pulmonary disease and asthma [1,3]. Furthermore, the genetic background has been associated with differences in human susceptibility to environmental agents including pesticides and infectious agents [4], and may be of importance also with regard to biological effects of particles.

Supporting a causal relationship between particles and increased allergic symptoms, particles have been shown to increase allergic responses both in humans and in animal models (reviewed in [5,6]). In rodent models, widely different particles have been shown to increase allergen-specific IgE levels both after inhalation, intratracheal, intranasal, intraperitoneal and subcutaneous exposure [7-12]. Particle characteristics like particle size, particle composition and chemicals adsorbed to the particle surface have been shown to influence the adjuvant effect on allergic sensitization and airway inflammation and hyperreactivity in mice [11-18]. For combustion particles, the insoluble particle core appears to play an important role in the adjuvant effect of particles in several mouse models [18-23]. Mouse strain differences in allergic airway inflammation and allergen-specific antibody levels have been reported after intratracheal instillation of diesel exhaust particles (DEP) and allergen [24,25]. Also with "clean" polystyrene particles (PSP, 0.1 μm diameter) as a surrogate of the insoluble combustion particle core, Granum *et al*. [26] concluded that the particle adjuvant effect differed qualitatively with regard to Th2- and Th1-associated antibody responses, depending on the genetic background or induced immune status of the mice.

The overall aim of the present study was to investigate how the genetic background influences the responses to the allergen and particles alone and in combination. We therefore examined the antibody and lymph node responses in various inbred strains of mice expected to display different responses to OVA and particles. BALB/cA mice are regarded as a high IgE responder strain to OVA [27,28]. NIH mice were included because they in an intraperitoneal model responded differently from BALB/cA mice to PSP and OVA [26]. Furthermore, we included C3H/HeN mice, which have been shown to be IgE low-responders to OVA [28]. BALB/cJ mice were included in the antibody experiments because this sub-strain has been reported to respond more strongly to OVA than BALB/cA mice in a subcutaneous model [29].

The lymph node is a key site in the priming of T lymphocytes and IgE production by B lymphocytes [11,30,31]. Therefore, in addition to serum antibody levels, we examined the primary cellular response in the draining popliteal lymph node (PLN) after footpad injection of polystyrene particles (PSP) and the allergen ovalbumin (OVA), alone and in combination. The PLN is the only lymph node draining the footpad [32], and is therefore well suited for studies of responses in the lymph node cells. We investigated how the selected inbred strains of mice differed qualitatively and quantitatively in their Th2- and Th1-associated serum antibody and PLN cellular response patterns to OVA+PSP. Furthermore, we investigated if the background (i.e. the *ex vivo *cytokine response in cells from mice injected with buffer only), OVA-, PSP- and OVA+PSP-induced cytokine patterns differed in the various mouse strains, and whether the cytokine patterns could explain the differences in the antibody responses to OVA+PSP. We found that the susceptibility to the adjuvant effect of particles on both secondary antibody responses and primary cellular responses was influenced by genetic factors (i.e. mouse strain). The *ex vivo *cytokine response patterns did not predict the *in vivo *antibody responses.

## Results

### The antibody adjuvant activity of PSP in different strains of mice

**Figure 1:**
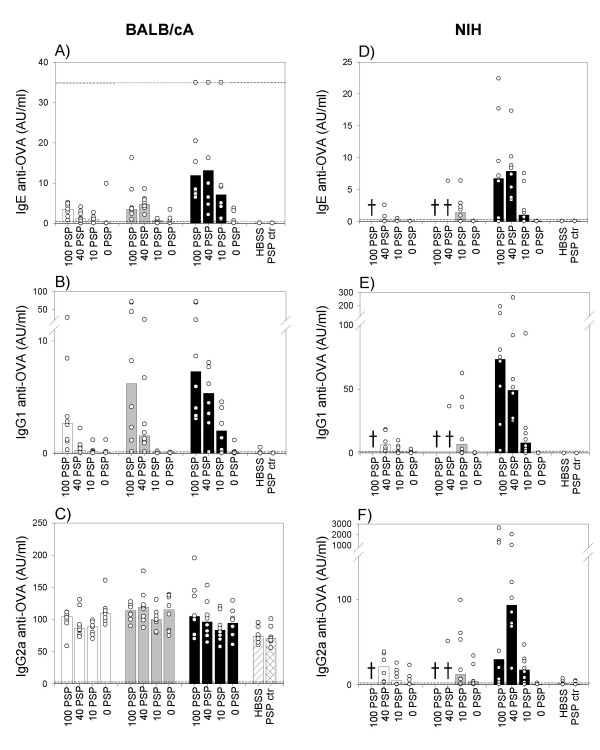
**Allergen and particle dose-response experiments in BALB/cA and NIH mice **Levels of serum OVA-specific IgE (A, D), IgG1 (B, E) and IgG2a (C, F) on day 26 after injection into one hind footpad of 100 (open columns), 50 (gray) or 10 (black) μg OVA combined with 100, 40, 10 or 0 μg PSP, or buffer (HBSS (hatched)), in BALB/cA (A, B, C) and NIH (D, E, F) mice. On day 21 the mice were boostered with the same OVA dose in the footpad. The PSP-control ("PSP ctr") group was given 100 μg PSP on day 0, followed by HBSS injection on day 21 (diamonds). Values (arbitrary units, AU) for individual mice (circles) and median values (columns) for groups of eight mice are shown. Dotted lines indicate the lower or higher detection limits for the ELISA assays. Note different scales for the two strains of mice.

To examine the dose-response relationship of OVA and PSP with regard to antibody production, BALB/cA and NIH mice were immunized with various dose combinations of OVA (100, 50 and 10 μg) and PSP (100, 40, 10 and 0 μg). After a booster dose of OVA on day 21, the OVA-specific IgE, IgG1 and IgG2a antibody levels in serum were determined on day 26. In BALB/cA mice, PSP given with OVA strongly increased the levels of the Th2-associated IgE and IgG1 antibodies compared to OVA alone (Figure 1A, B). In contrast, the Th1-associated IgG2a levels did not differ between the groups given OVA in the presence or absence of PSP (Figure 1C). Overall, there was an inverse relationship between the OVA dose and both the IgE and IgG1 levels, with 10 μg of OVA giving the highest antibody levels for all particle doses. Analyses of the factorial design showed that the dose of 10 μg OVA, regardless of PSP dose, induced significantly higher IgE levels than the other OVA doses, and higher IgG1 levels than 100 μg OVA (p < 0.001). Irrespective of OVA dose, the IgG1 response increased significantly with each increase in PSP dose (p < 0.001). A dose-response trend for particles was less clear for IgE due to an absence of a difference between 40 and 100 μg PSP.

In NIH mice, PSP also exerted an adjuvant effect on the antibody responses to OVA, however NIH mice responded differently from BALB/cA mice in two ways. First, the NIH mice receiving the highest dose combinations of OVA and PSP experienced anaphylactic reactions and died within minutes after the OVA booster injection (indicated with crosses in Figure 1D, E, F). Secondly, also the OVA-specific IgG2a levels were increased by PSP in NIH mice, in contrast to BALB/cA mice. However, the responses in the surviving mice (i.e. 10 μg OVA) suggest that IgE and IgG1 responses were similar in NIH and BALB/cA mice with regard to the dose-response relationship for particles. Also the IgG2a levels were significantly higher for OVA with all particle doses than for OVA alone, and significantly higher antibody levels were observed with 40 μg PSP than with 10 μg PSP.

**Figure 2:**
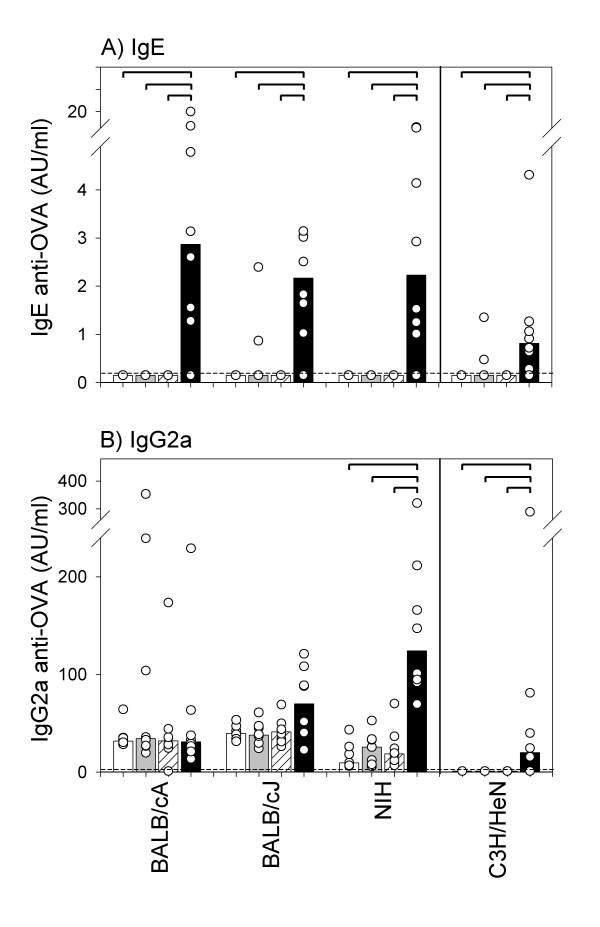
**The adjuvant effect of PSP on OVA-specific IgE and IgG2a responses in different strains of mice **Levels of serum OVA-specific IgE (A) and IgG2a (B) on day 26 after injection into one hind footpad of HBSS (open columns), 10 μg OVA (gray), 40 μg PSP (hatched) or 10 μg OVA + 40 μg PSP (black) in different mouse strains. On day 21 all mice were boostered with 10 μg OVA in the footpad. Vertical straight lines indicate separate experiments. Values (arbitrary units, AU) for individual mice (circles) and median values (columns) for groups of mice are shown. Dotted lines indicate the lower detection limits for the ELISA assays. Brackets indicate statistically significant differences between groups (p < 0.05). All experiments were performed twice with similar results, with eight mice per group.

As 10 μg OVA and 40 μg PSP gave a medium but still pronounced response, these doses were used in further experiments. Next, we compared the allergen-specific antibody responses after immunization with OVA+PSP, OVA and PSP in BALB/cA, BALB/cJ, NIH and C3H/HeN mice. As shown in Figure 2, the response patterns for BALB/cA and NIH mice were similar to those shown in Figure 1, with increased IgE production in both strains, whereas IgG2a production was increased only in NIH mice. Furthermore, in our system, BALB/cJ mice had specific IgE and IgG2a responses similar to BALB/cA mice, however the IgG2a levels tended to be slightly increased by OVA+PSP in BALB/cJ mice (statistically significant in one of two experiments). Similar to the responses in NIH mice, PSP had an adjuvant effect on both IgE and IgG2a responses in C3H/HeN mice, although the responses appeared weaker in C3H/HeN than in NIH and BALB/c mice.

Immunization with OVA alone gave IgE responses in some individual BALB/cA, BALB/cJ and C3H/HeN mice, but never in NIH mice (Figure 1 and 2). As expected, HBSS and PSP without allergen did not affect the specific antibody levels in any of the strains.

### Cell numbers and *ex vivo *cytokine responses in the draining lymph node during the primary response

To examine how the primary cellular response to OVA and PSP, alone or in combination, varied in BALB/cA, NIH and C3H/HeN mice, we studied the cell numbers and the *ex vivo *cytokine secretion by the popliteal lymph node (PLN) cells. Five days after injection of OVA+PSP, OVA, PSP and buffer (HBSS) into the footpad, the PLN cell numbers were determined, and the amount of IL-4, IFN-γ and IL-10 released from PLN cells after Con A stimulation *ex vivo *were determined.

**Figure 3:**
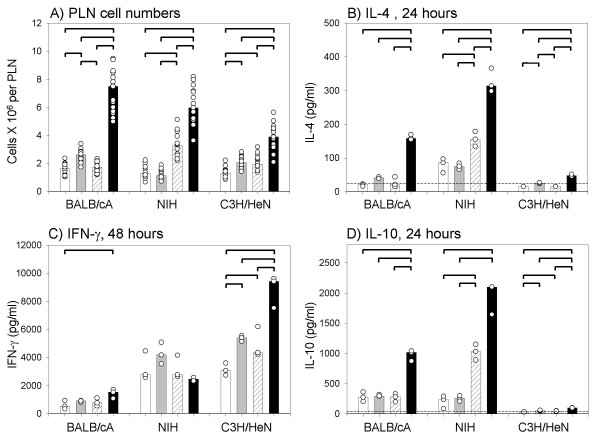
**The primary cellular response in the draining lymph node **The primary cellular response in the draining PLN was determined five days after a single injection of HBSS (open columns), 10 μg OVA (gray), 40 μg PSP (hatched) or 10 μg OVA + 40 μg PSP (black) into both hind footpads of BALB/cA, NIH and C3H/HeN mice. The total lymph node cell numbers (A) and IL-4 (B), IFN-γ (C) and IL-10 (D) secreted from the PLN cells after *ex vivo* stimulation with Con A were determined. Values for individual samples (circles) and group median values (columns) are shown. Dotted lines indicate the lower detection limits for the cytokine ELISA assays, and brackets indicate statistically significant differences between groups (p < 0.05).

For all three strains, the PLN cell numbers were significantly increased by OVA+PSP compared to all three control groups (Figure 3A). Interestingly, the cell number response to OVA alone and PSP alone (compared to HBSS) varied between the three strains. In BALB/cA mice, OVA significantly increased the cell numbers, whereas PSP significantly increased the cell numbers in NIH mice. C3H/HeN mice took an intermediate position, as both OVA and PSP weakly but significantly increased the cell numbers compared to HBSS.

With regard to the cytokines (Figure 3B, C and 3D), OVA+PSP strongly increased the levels of IL-4 and IL-10 compared to the three control groups in BALB/cA mice (p < 0.006), whereas the IFN-γ levels were only marginally affected (p < 0.035). Furthermore, OVA tended to weakly (n.s.) increase the IL-4 levels in BALB/cA mice. The IL-4 levels for the BALB/cA control groups were relatively low compared to levels in NIH mice. In NIH mice, OVA induced no changes in cytokines or cell numbers, whereas PSP significantly increased the IL-4 and IL-10 levels (p < 0.007), and OVA+PSP also increased these "responsive" cytokines (p < 0.005, compared to OVA). The IFN-γ levels in NIH mice were not altered by PSP. Again, the C3H/HeN mice responded to both OVA and PSP. All cytokines were significantly increased by OVA and further increased by OVA+PSP in C3H/HeN mice, although more pronounced for IFN-γ than for IL-4 and IL-10. PSP alone also significantly increased the levels of IFN-γ and IL-10 in C3H/HeN mice. The levels of IL-4 and IL-10 were low and the levels of IFN-γ were high in C3H/HeN mice compared to the levels in the other strains. The background levels (buffer treated animals) of IL-4 and IFN-γ were relatively low in BALB/cA and relatively high in NIH mice, whereas the levels in C3H/HeN mice appeared low for IL-4 and high for IFN-γ, respectively.

## Discussion

The genetic background appears to strongly influence whether an individual develops allergic immune responses to an allergen [33]. In mice, this is illustrated by reports of strain differences in allergic responses to allergens [28,34-36]. In agreement with Granum *et al*. [26], our data indicate that also the antibody-enhancing capacity of the "clean" insoluble particle is influenced by the genetic background. This is consistent with reports of mouse strain differences in allergic airway inflammation and specific antibody levels after intratracheal instillation of DEP and allergen [24,25]. Also the effects of particles on airway inflammation, epithelial permeability and alveolar macrophage phagocytic activity have been shown to vary between mouse strains [37].

The dose-response relationship for particles appeared similar for BALB/cA and NIH mice, as the low OVA dose (10 μg) and the high particle doses (40 and 100 μg PSP) induced the strongest IgE and IgG1 antibody responses. However, the NIH mice immunized with a high concentration of both OVA and PSP were lost due to anaphylactic shock after the booster injection. High booster antigen concentrations have earlier been shown to induce anaphylactic shock in mice after subcutaneous priming with horse gamma globulin covalently bound to cellulose particles [38]. In mice, IgG(1) has been suggested to be as effective as IgE in triggering anaphylactic reactions [39,40]. The induced levels of OVA-specific IgG1 appeared to be about 10 times higher in the surviving NIH mice than in BALB/cA mice (Figure 1B and 1E). Thus, NIH mice may be more susceptible than BALB/cA mice to experience anaphylactic shock either because of a stronger IgG1 response, or because of an otherwise greater susceptibility to anaphylactic shock.

In the present study, the antibody adjuvant effect of PSP differed between the different strains of mice, both with regard to the magnitude and whether a Th2 or a mixed Th1- and Th2-associated antibody response was increased. Whereas the Th1-associated IgG2a response was markedly increased by PSP only in NIH and C3H/HeN mice, PSP enhanced the Th2-associated IgE response in all mouse strains examined. PSP seemed also to increase IgE responses in pilot studies with C57BL/6J and C3H/HeJ mice (data not shown). Also with regard to the adjuvant effect of PSP, the BALB/cA mice appeared to be a high IgE-responder, whereas the C3H/HeN mice seemed to be a relatively low responder for both OVA-specific IgE and IgG2a antibody levels. This responder status is in accordance with other studies of the IgE responsiveness to OVA in these strains of mice [27,28].

Also with regard to the primary cellular responses in the lymph node, PSP increased the response to OVA in both BALB/cA, NIH and C3H/HeN mice (Figure 3). However, while IL-4 and IL-10 cytokine responses were significantly increased by OVA+PSP in all three strains, in C3H/HeN mice the levels were low and the increases may not be biologically significant. On the other hand, OVA+PSP markedly increased the IFN-γ levels in C3H/HeN mice, and marginally in BALB/cA mice. IL-4 and IFN-γ cytokines are often used as indicators for a Th2 and Thl response, respectively [41]. In this regard, the cytokine responses to OVA+PSP could be characterized as Th2-, Th2- and Th1-biased for BALB/cA, NIH and C3H/HeN mice, respectively, which would not, however, predict the balance between Th2- and Th1-associated antibody responses (Figure 1 and 2, see below). Neither could the background (buffer control) responses for IL-4 and IFN-γ in the different strains explain the balance between Th2- and Th1- associated antibody responses to OVA+PSP.

The complexity in gene-environment interactions were illustrated by the highly varying responsiveness to OVA and PSP given separately in the three strains examined. BALB/cA mice responded with a non-significant but consistent increase in IL-4 cytokine release in OVA-treated mice, whereas NIH mice responded with a significantly increased Th2-biased (IL-4 and IL-10) cytokine release in PSP-treated mice. C3H/HeN mice responded with both Th1- and Th2-type cytokine secretion, although apparently Th1-skewed due to low IL-4 and IL-10 levels, after both OVA or PSP treatments. This suggests that the genetic background strongly influences the susceptibility to immunogens and irritative stimuli such as particles, also with regard to the Th1 and Th2 cytokine balance of the response. Interestingly, PSP alone increased the Th2-associated, but not the Th1-associated, cytokine responses in NIH mice. If particles by themselves are able to induce IL-4 production in the lymph nodes also *in vivo*, this might promote an allergic response to the allergen present.

Taken together, PSP exerted an adjuvant effect on OVA-induced IgE and IL-4 levels in all three strains, while there was no association between the Th1-associated parameters IgG2a and IFN-γ. Thus, for the strains of mice used, the primary *ex vivo *cytokine pattern could not predict the Th2- and Th1-associated antibody pattern. One reason could be that the Con A-stimulation *in vitro *was not an optimal way to describe the actual *in vivo *cytokine secretion by the PLN cells. However, *in vitro *OVA-stimulation of PLN cells from OVA+PSP treated BALB/cA mice gave similar IL-4 and IFN-γ response pattern as Con A-stimulation (data not shown). Our findings are consistent with previous studies showing that cytokine expression was only in part correlated with airway disease and IgE responses [35,42], and our data illustrate that interpretation and extrapolation of *in vitro *data should be done with caution. The description of the Th2/Th1 balance may explain parts of an immune response, however the immune regulation is more complex, involving other cytokines, cell surface molecules and regulatory T cells. IL-10 seems to play an important role in the development and function of certain regulatory T cells (reviewed in [43,44]). However, in the present study, IL-10 secretion by the pool of PLN cells was highly correlated with the secretion of IL-4 (r_s _= 0.86). Although contribution from regulatory T cells could not be excluded, this indicates that IL-10, which are secreted to a higher degree by Th2 than Th1 cells [45], in the PLN at this stage was produced mainly by Th2 cells.

In agreement with Randolph et al. [46], we have previously shown that both PSP, DEP and ambient air particles in the draining lymph node were located within acid phosphatase-, MHC class II- and CD86-positive cells, most probably antigen-presenting cells (APC) [23,47]. It is remarkable that PSP, in the absence of allergen, affected the lymph node cells in both NIH and C3H/HeN mice. In contrast, our present and previous data from BALB/cA mice indicate that although the particles synergistically enhanced the cellular responses to OVA, model particles (PSP, DEP) had no detectable effects neither on PLN cell numbers, expression of lymphocyte surface molecules nor on *ex vivo *cytokine secretion by the PLN cells [23,47]. Taken together, our data from BALB/cA mice therefore suggest a role for solid particles as allergen carriers [48] in the adjuvant effect on allergic sensitization. Our present data suggest that particles, in susceptible strains, additionally influence the immune response in the lymph node. In our model it appears that the particles which induced cellular responses in the absence of allergen (i.e. ambient air particles in BALB/cA mice [47] as well as PSP in NIH and C3H/HeN mice (present study)) enhanced both Th2- and Th1-associated antibody responses, whereas the particles enhancing a Th2-dominated antibody response (i.e. PSP and DEP in BALB/cA mice) did not induce detectable cellular changes themselves. This suggests that the insoluble particle core act by enhancing the inherent response to the allergen (acts like a "general motor" [6]), and that genetic background and particle properties determine whether an additional Th1-associated response is elicited. The presence of a Th1 response has been suggested to amplify Th2-driven inflammation [49-52]. Our results therefore suggest that particles may both enhance the IgE sensitization and, depending on genetically determined susceptibility, further aggravate allergic inflammation and thereby increase the allergic symptoms in some individuals.

## Conclusion

PSP enhanced the OVA-specific IgE antibody responses in all strains of mice examined, whereas the IgG2a response was increased to varying degrees and only in some strains. Genetic factors (i.e. the strain of mice) influenced the responsiveness to the adjuvant effect of PSP on secondary antibody responses and the primary cellular responses to OVA. Furthermore, genetic factors also influenced the lymph node cell responsiveness to both OVA and PSP given separately. Interestingly, PSP alone induced different cytokine responses in the lymph node in some of the mouse strains. Furthermore, we found that the *ex vivo *cytokine patterns could not predict the *in vivo *Th2- and Th1-associated antibody response patterns in the different mouse strains. The results indicate that the insoluble particle core acts by increasing the inherent response to the allergen, and that the genetic background may determine whether an additional Th1-associated component is added.

## Methods

### Animals

Female inbred BALB/cABomTac and BALB/cJBomTac mice (Taconic M&B A/S, Ry, Denmark) and NIH/OlaHsd and C3H/HeNHsd mice (Harlan UK Ltd., Oxon, UK) were used. The mice were housed, 5–8 animals per cage, on BeeKay bedding (B&K Universal AS, Nittedal, Norway) in type III macrolon cages in filter cabinets (Scantainers). The animals were allowed to rest for at least one week before entering the experiments 6–7 weeks old. The mice were exposed to a 12-hr/12-hr light/dark cycle (30–60 lux in cages), room temperature (20 ± 2°C) and 40–60% relative humidity. The animals were given pelleted food (RM1, SDS, Essex, UK) and tap water *ad libitum*. The experiments were performed in conformity with the laws and regulations for experiments with live animals in Norway, and they were approved by the Experimental Animal Board under the Ministry of Agriculture in Norway.

### Particles and allergen

As a surrogate for the insoluble particle core of combustion particles, polystyrene particles (PSP) with a diameter of 0.1 μm were used (Polybead Polystyrene Microspheres; Polysciences Europe GmbH, Eppelheim, Germany). As allergen, ovalbumin (OVA, Gal d 2; chicken egg albumin, grade VII, Sigma, St. Louis, MO, USA) was used. In the dose-response experiments, we used particle doses of 100, 40 and 10 μg per injection (14.9, 5.94 and 1.49 × 10^10 ^particles, respectively), as well as OVA doses of 100, 50 and 10 μg per injection. For all further studies, the dose of 40 μg PSP (5.94 × 10^10 ^particles) and 10 μg OVA per injection was used. All preparations were made in Hank's balanced salt solution (HBSS; PAA Laboratories GmbH, Linz, Austria). 20 μl of the different preparations were injected subcutaneously into the hind footpad (heal-toe direction) using a 100 μl Hamilton syringe (Hamilton Bonaduz AG, Switzerland) with a 30 G sterile needle (BD Medical Systems, Ireland).

### Experimental design

Dose response experiments were performed in groups of eight BALB/cA or NIH mice, with all combinations of 100, 40, 10 and 0 μg PSP and 100, 50 and 10 μg OVA. The suspensions were injected into the right hind footpad on day 0, and a booster with the same OVA dose was given on day 21. On day 26, the mice were exsanguinated by heart puncture under CO_2 _anesthesia. Serum was stored at -20°C until analyzed. Serum levels of OVA-specific IgE, IgG1 and IgG2a were measured by enzyme-linked immunosorbent assay (ELISA). Next, to further examine the qualitative differences in the adjuvant effect of particles, antibody responses after injection of OVA+PSP, OVA, PSP and buffer (HBSS) were determined in the mouse strains NIH, BALB/cA and BALB/cJ, and in a separate experiment in C3H/HeN. These experiments were all performed twice, with eight mice per group.

In a model previously described [23], we studied the primary cellular response in the draining lymph node in BALB/cA, NIH and C3H/HeN mice (fifteen mice per group). The popliteal lymph nodes (PLN) were excised five days after injection into both hind footpads of OVA and PSP, separately or in combination. After pooling of the PLN from the same animal, the total cell numbers per PLN were determined (Coulter Counter). Thereafter, PLN cells from five animals were pooled to achieve sufficient cell numbers per well for *ex vivo *culture with Con A, and the cytokine concentrations in cell culture supernatants were determined by sandwich ELISAs. In one set of experiments, the responses of cells from OVA and OVA+PSP treated animals from all strains were determined, and untreated mice were studied in a separate experiment. A confirming set of experiments was then conducted, this time performed in separate experiments for each strain of mice (the results shown in Figure 3).

### ELISA for detection of IgE, IgG1 and IgG2a anti-OVA antibodies

IgE, IgG1 and IgG2a anti-OVA antibodies were detected in a capture ELISAs as previously described [19,53], as modified in [54]. In short, microtiter plates coated with a monoclonal rat anti-mouse IgE, rat anti-mouse IgG1 or rat anti-mouse IgG2a antibody were incubated with the mouse sera in triplicates, diluted 1:10, 1:20 and 1:20, respectively. After incubation with biotinylated OVA, followed by incubation with pre-formed complexes of streptavidin and biotinylated alkaline phosphatase, *p*-nitrophenyl phosphate disodiumhexahydrat diluted in a diethanolamine buffer was added as substrate. Dilutions of a standard mouse OVA-immune serum pool were included on each plate for standard curve production. OD was measured at 405 nm on a MRX Microplate Reader (Dynatech Laboratories, Chantilly, VA, USA) connected to a PC using BioLinx software (Dynatech Laboratories), and the antibody concentrations are given in arbitrary units (AU) per ml.

### PLN single cell suspensions and *ex vivo *cytokine secretion after Con A stimulation

Five days after injection, the popliteal lymph nodes were excised and single cell suspensions prepared as previously described [23]. After pooling of the two PLN cell suspensions from the same animal, the cell concentration was measured with a Coulter Counter Z1 (Beckman Coulter Incorporated, FL, USA), and presented as the total cell number (10^6^) per PLN. Thereafter, as described elsewhere [23], the PLN cells were cultured with 5 μg/ml Concanavalin A (Con A, Sigma, MO, USA), and *ex vivo *IL-4, IFN-γ and IL-10 cytokine secretion into the culture supernatants was determined by Mouse DuoSets ELISA (R&D Systems Inc., MN, USA). The optimal incubation times for the measurement of IL-4, IFN-γ and IL-10 with BALB/cA mice were previously found to be 24, 48 and 24 hours, respectively [23], and data for these time points are presented.

### Statistical analysis

Statistical analysis was performed with SigmaStat ^® ^Statistical Analysis System for Windows Version 2.03 (Jandel Scientific, Erkrath, Germany). Antibody levels, cell numbers and cytokine levels were log_10_-transformed to achieve normally distributed data with equal variances. In the dose-response experiments in BALB/cA mice, the data were analyzed in a two way ANOVA, using "OVA dose" as factor 1 and "PSP dose" as factor 2. Due to lost animals in the experiment with NIH mice, similar analyses could not be performed, and a one way ANOVA was run only on the data with 10 μg OVA. In both strains, the pair-wise comparisons were performed using Tukey's post hoc test. In all other experiments, to assure equal criteria for all strains independent of experimental size/setup, the data from each strain of mice were analyzed separately, using a parametric one way ANOVA. If the ANOVAs showed statistical differences with a significance of p ≤ 0.05, pair-wise comparisons of all groups within each mouse strain were performed by Tukey's post hoc test. Experimental groups were considered statistically different if p ≤ 0.05, and differences found statistically significant in two experiments are indicated in the figures.

## Authors' contributions

UCN carried out the experimental work, statistical analyses and wrote the manuscript. ML initiated the project and obtained funding. UCN, ML and AA participated in designing the study and the interpretation of the data, and edited the manuscript. All authors read and approved the final manuscript.
